# Exploring the Capability of Yeasts Isolated from Colombian Fermented Cocoa Beans to Form and Degrade Biogenic Amines in a Lab-Scale Model System for Cocoa Fermentation

**DOI:** 10.3390/microorganisms9010028

**Published:** 2020-12-24

**Authors:** Johannes Delgado-Ospina, Laura Acquaticci, Junior Bernardo Molina-Hernandez, Kalliopi Rantsiou, Maria Martuscelli, Astride Franks Kamgang-Nzekoue, Sauro Vittori, Antonello Paparella, Clemencia Chaves-López

**Affiliations:** 1 Faculty of Bioscience and Technology for Food, Agriculture and Environment, University of Teramo, Via R. Balzarini 1, 64100 Teramo, Italy; jbmolinahernandez@unite.it (J.B.M.-H.); mmartuscelli@unite.it (M.M.); apaparella@unite.it (A.P.); 2 Grupo de Investigación Biotecnología, Facultad de Ingeniería, Universidad de San Buenaventura Cali, Carrera 122 # 6-65, Cali 76001, Colombia; 3 School of Pharmacy, University of Camerino, Via Sant’ Agostino 1, 62032 Camerino, Italy; laura.acquaticci@unicam.it (L.A.); astride.kamgang@unicam.it (A.F.K.-N.); sauro.vittori@unicam.it (S.V.); 4 Department of Agricultural, Forestry and Food Sciences, University of Turin, Largo Paolo Braccini 2, 10095 Grugliasco, Torino, Italy; kalliopi.rantsiou@unito.it

**Keywords:** biogenic amine, volatile compounds, organic acid, fermentation, cocoa

## Abstract

Yeast starters for cocoa fermentation are usually tested according to their enzymatic activities in terms of mucilage degradation and flavor improvement, disregarding their influence on the production or elimination of toxic compounds as biogenic amines (BAs), important for human health. In this work, we tested 145 strains belonging to 12 different yeast species and isolated from the Colombian fermented cocoa beans (CB) for their capability of producing BAs in vitro. Sixty-five strains were able to decarboxylate at least one of the amino acids tested. *Pichia kudriavzevii* ECA33 (Pk) and *Saccharomyces cerevisiae* 4 (Sc) were selected to evaluate their potential to modulate BAs, organic acids, and volatile organic compounds (VOCs) accumulation during a simulated cocoa fermentation. The growth of *Sc* or *Pk* in the presence of CB caused a significant reduction (*p* < 0.05) of 2-phenylethylamine (84% and 37%) and cadaverine (58% and 51%), and a significant increase of tryptamine and putrescine with a strong influence of temperature in BA formation and degradation. In addition, our findings pointed out that *Pk* induced a major production of fatty acid- and amino acid-derived VOCs, while *Sc* induced more VOCs derived from fatty acids metabolism. Our results suggest the importance of considering BA production in the choice of yeast starters for cocoa fermentation.

## 1. Introduction

Cocoa beans fermentation is driven by a complex of microorganisms that accomplish their activity in a well-defined chronological succession of yeasts, lactic acid bacteria (LAB), acetic acid bacteria (AAB), *Bacillus*, and filamentous fungi. These microbial activities are important not only to the degradation of the mucilage but also for the production of molecules that are precursors of the distinctive aroma, flavor and color, which are formed during drying and roasting [1].

The functional role of the different microbial groups that ferment cocoa beans are already established, and in particular yeast growth and activity, which is crucial for effective cocoa bean fermentation to obtain the desired sensory properties of cocoa. Due to their pectinolytic activities, their tolerance to ethanol, acid and heat, as well as to their adaptation to the nutritional environment of the fermenting cocoa pulp-bean mass, yeasts dominate during the first step of fermentation. The main impact of yeasts to the cocoa beans fermentation process is due to the alcoholic fermentation of the pulp sugars and pectin degradation that facilitates bean aeration, therefore favoring bacterial development, and starting the second phase of fermentation [2]. Moreover, they contribute to the development of the aroma precursors important to the formation of sensory profiling for cocoa beans [3] by different enzyme activities [4]. One of the principal concerns in cocoa quality reduction is a slow fermentation and the excessive production of lactate and ethanol, which are consequences of an excess of seed pulp, yielding more nutrients (e.g., excess of fermentable carbohydrates) and less oxygen dispersion in the fermenting beans [5]. Another less studied concern regarding cocoa fermentation is the production of biogenic amines (BAs) during the process, which has harmful effects on cocoa quality and human health [6]. In particular, histamine (HIS) causes headaches, low blood pressure, heart palpitations, oedema, vomiting, diarrhoea, and other symptoms [7]. In addition, the assumption of tyramine (TYR) and phenylethylamine (PHE) contribute to an increase in arterial pressure. Furthermore, although putrescine (PUT) and cadaverine (CAD) are not toxic themselves, they can interfere with the enzymes that metabolize BAs, intensifying their effects [8].

During fermentation, cocoa proteins can be hydrolyzed by aspartic endoprotease and carboxypeptidase, two enzymes naturally present in cocoa beans, to release free amino acids (FAA), which once released can be subjected to the activity of microbial decarboxylases, leading to BAs formation [9]. In addition, naturally occurring microorganisms such as yeasts, filamentous fungi, LAB and AAB, are able to hydrolyze the proteins present in cocoa beans, thus providing FAA to the BA synthesis [10] or the production of fusel alcohols, acids and esters. Although many factors influence decarboxylase activity, this is amplified at low pH values, as a protection mechanism of bacteria against the acid medium [11]. In general, LAB is the most important biogenic amine producers in fermented foods [9], in spite of other less studied microorganisms like *Enterobacteriaceae*, *Pseudomonads*, *Clostridium perfringens* and yeasts.

It is well established that starter cultures could be a feasible opportunity for obtaining a safe fermentation process that generates the desirable characteristics of chocolate. Thus, the possibility of using yeasts as starter cultures for cocoa fermentation was suggested due to their positive features for cocoa. *Saccharomyces cerevisiae*, *Kluyveromyces marxianus*, *Pichia kluyveri*, *Torulaspora delbrueckii*, and *P. kudriavzevii* dominated in most studies on yeast starters associated with cocoa fermentation [2,5,12,13,14,15,16]. Although the studies mentioned above had shown the possibility of achieving good results in terms of mucilage degradation and sensory quality, there is no specific study on the influence of yeasts isolated from cocoa fermented beans on the BAs behaviour.

Since the selective pressure of tropical environments may favor microbial biodiversity with a useful technological potential [17], speciality cocoa can be produced by promoting the growth of some particular microbial groups during fermentation or by using starters. The first aim of this study was to evaluate the ability of 145 yeast strains isolated from Colombian fermented cocoa and dried beans, to decarboxylate selected amino acids involved in BA production, in particular those involved in the production of CAD, HIS, spermidine (SPD), and spermine (SPM), the main BAs formed during cocoa production [6]. The second aim of this study was to assess the technological potential of two selected yeast strains of *P. kudriavzevii* ECA33 and *S. cerevisiae* 4, by determining their ability to induce the BAs formation or degradation during cocoa fermentation and to modulate the organic acids and volatile organic compounds (VOCs) constituents during a lab-scale cocoa beans fermentation.

## 2. Materials and Methods

### 2.1. Strains

In this study, we used strains belonging to the species *Candida parapsilosis* (10) *Hyphopichia burtonii* (8), *Pichia kudriavzevii* (54), *Pichia manshurica* (13), *S. cerevisiae* (38), *Schizosaccharomyces pombe* (4), *Starmerella sorbosivorans* (2), *Torulaspora delbrueckii* (2), *Torulaspora pretoriensis* (2), *Trichosporon asahii* var. asahii (3), *Wickerhamomyces anomalus* (6), and *Zygosaccharomyces bisporus* (2). These strains belonging to the culture collection of the Faculty of Bioscience and Technology for Food, Agriculture and Environment of the University of Teramo, Italy, were previously isolated from Colombian cocoa beans and identified by molecular methods [18].

### 2.2. Screening for Amino Acid Decarboxylation

To evaluate the capability of yeast strain cells to decarboxylate amino acids, producing biogenic amines, the medium reported by [19] was used. The tested amino acids were histidine (HIS), tyrosine (TYR), lysine (LYS), phenylalanine (PHE), and ornithine (ORN). We performed a qualitative screening focus to evidence the potential of the yeast isolated to produce BA at 30 and 37 °C, utilising 96-well microtitre plates, incubated at 30 °C and 37 °C for 8 days. The activity was evidenced by the variation of the medium color from yellow to purple. The activity was visually classified according to the intensity of purple color on a scale from 0 (no-change in color) to 3 (very intense color).

### 2.3. Cocoa Samples

Cocoa pods of the Colombian Criollo variety were harvested from plantations in three regions of Colombia: Valle del Cauca, Cauca, and Nariño [6]. The samples were transported to the University of San Buenaventura Cali, Colombia. The pod samples were kept at 4 °C for one day, then pods were opened and beans were removed using a knife. The beans were uniformly mixed, freeze-dried by a lyophilizer (FreeZone 4.5 L, Labconco, KS, USA), and finally stored at 4 °C until their use.

### 2.4. Lab-Scale Fermentation

Thirty-six–hour old cells of *S. cerevisiae* 4 or *P. kudriavzevii* 33A were used as the individual native starter. Yeast cells cultivated in Yeast Peptone Dextrose (YPD) and incubated at 30 °C were centrifuged (5000× *g*, 10 min, 4 °C), washed twice in 50 mM sterile potassium phosphate buffer (pH 7.0), and successively re-suspended to a final optical density at 620 nm of 0.7 to reach a final cell concentration of about 10^6^ CFU/mL. The inoculum amount was confirmed plating an aliquot on YPD agar incubated at 30 °C for 48 h.

To rehydrate the dried beans, we used ultrapure water, until the moisture content reached 35%, successively beans were mixed with simulated cocoa pulp liquid medium (SCPM) as reported by [20], with modifications mainly regarding the quantity of the SCPM, in particular, we used rehydrated beans (5000 g) mixed with 1500 mL of SCPM. Successively, 10 mL of yeast suspension were inoculated singularly in 450 g of unfermented cocoa beans, placed in 1000 mL of plastic fermentation containers that were covered with lids and subjected to a controlled temperature, mimicking the conditions observed during on-farm fermentation as follows: for fermentation, the containers were incubated at 25 °C (0–12 h), 30 °C (12–24 h), 35 °C (24–36 h), 40 °C (36–48 h), 45 °C (48–72 h), and 48 °C (72–144 h) to mimic the temperature changes occurring through the cocoa fermentation as reported by [2] in a chamber under 95% humidity. Samples were aseptically taken at 24, 48, 72, and 120 h, analyzed immediately for microbial analyses and stored at −20 °C for the analysis of BA, organic acids, and volatile compounds content. All experiments were performed in triplicate with a total of 9 batches.

### 2.5. pH Determination

Periodically, 10 g of samples were shaken in 100 mL of MiliQ water for 15 min, after bean separation pH was measured in the supernatant using a pH meter (FE20, Mettler Toledo, Columbus, OH, USA).

### 2.6. Microbiological Analyses

Ten grams of fermented cocoa bean sample, for each time or period of laboratory-scale fermentation, were subjected to 1:10 serial dilution, and the enumeration of cells was performed via plate count on YPD agar (Oxoid, Waltham, MA, USA) containing 100 mg/L of chloramphenicol, incubated at 25 °C for 3–4 days. LAB was enumerated on de Man Rogosa Sharpe (MRS) Agar (Oxoid, Waltham, MA, USA) containing 100 mg/L of cycloheximide at 30 °C for 3–4 days. Plates were incubated under modified atmosphere (76% N_2_, 4% O_2_, and 20% CO_2_). For the AAB enumeration, GYC (5% glucose, 1% yeast extract, 0.5% CaCO_3_, and 2.0% agar) supplemented with 7% ethanol and 100 mg/L of cycloheximide was used, incubating plates at 30 °C for 4–5 days. Plates were incubated in aerobiosis. Plate count was carried out in duplicate and results were expressed as mean and standard deviations of Log CFU/g. Ten colonies from the highest dilution plates for the presumptive LAB and AAB were isolated, purified and subjected to preliminary tests as morphology, Gram, catalase, oxidase, mobility, and acidification.

### 2.7. Determination of Biogenic Amines

Sample preparation was performed according to the methods reported by [11] with slight modifications. Briefly, one gram of sample was defatted through three successive washes with 5 mL of hexane (3 × 5 mL), and then defatted samples were dried under nitrogen. The extraction of BAs was performed with 10 mL (5 mL × 2) of trichloroacetic acid (TCA) under sonication at 59 kHz for 20 min. Samples were centrifuged at 5000 rpm for 10 min and supernatants were combined and filtered with filter paper. After extraction, BAs were derivatized and analysed following a method proposed by [21]. Briefly, 1 mL of the extract was mixed with 100 µL of 1,7-diaminoheptane (10 µg/mL), 300 µL of a saturated solution of NaHCO_3_, 50 µL of NaOH (2M), and 2 mL of dansyl chloride solution (10 mg/mL in acetone). Derivatization was performed in dark conditions at 45 °C for 45 min. The derivatized samples were then purified through a Strata C18-E cartridge (6 mL, 1 g). The eluate was filtered on 0.45 µm PTFE filter for analysis in HPLC-DAD.

### 2.8. Organic Acids Extraction and Determination

Organic acids were extracted following the procedure established by [22]. Three grams of cocoa bean sample were homogenized with 15.0 mL of deionized water for 20 s, by using a vortex (Mix, FALC Instruments, Treviglio, (BG) Italy). At this point, the homogenized compound was centrifuged (Mega Star 3.0, VWR International, Radnor, PA, USA) at 8000 rcf for 45 min, at 25 °C. Then, the supernatant was separated, and pH was adjusted to 8–9 using a 5 N NH_4_OH solution. Organic acids were separated by using a High-Performance Liquid Chromatography (HPLC) (Perkin Elmer Series 200, Waltham, MA, USA) equipped with an autosampler and provided with an ion-exchange column Biorad Organic acid 300 × 8 mm, 8 µm (Biorad Laboratories, Hercules, CA, USA). The column operated at 45 °C. 10 µL of the extract were injected into the chromatographic system. The mobile phase consisted of 4.5 mM sulphuric acid; the flow rate of 0.4 mL/min. The absorbance of the eluted sample was monitored by a UV detector, set at 210 nm. Identification of compounds was carried out considering the retention times of organic acid standards (acetic acid, citric acid, formic acid, galacturonic acid, lactic acid, pyruvic acid and succinic acid). Quantification was performed by calibration curves for each organic acid.

### 2.9. Determination of Volatile Organic Compounds (VOCs)

Solid Phase Micro-Extraction coupled with Gas Chromatography-Mass Spectrometry (SPME/GC-MS) was used to determined VOCs as reported by [23,24] with some modifications. The fibre used for SPME was coated with a Divinylbenzene/Carboxen/Polydimethylxilosane (DIV/CAR/PDMS) 50/30 μm thickness, and for VOCs extraction, we used 3 g of samples that were heated at 50 °C for 40 min to reach the equilibrium and for further 30 min to the VOCs fibre absorption.

Identification of the peaks was carried out by computer matching of mass spectral data with those of the compounds contained in the Agilent Hewlett-Packard NIST 98 and Wiley version 6 mass spectral database. The volatile compounds content was expressed as a relative percentage area.

### 2.10. Statistical Analyses

Results obtained were subjected to a statistical analysis using XLSTAT software version 2019.1 for Microsoft Excel (Addinsoft, New York, NY, USA). Pearson correlation among the amounts of BAs of the three batches and temperature of fermentation at different times was determined for each BA. One-way analysis of variance (ANOVA) was conducted. Tukey’s test was applied to compare the significance of differences between mean values at a significance level of α = 0.05. Hierarchical clustering analysis (HCA) and heatmap were performed using ClustVis web server [25].

## 3. Results

### 3.1. Screening of Biogenic Amines (BAs) Production

In this first part of the work, we characterized 145 yeast strains for their ability to decarboxylate some amino acids in vitro. A high percentage of the strains (45%) decarboxylated amino acids, without showing significant differences when incubated at 30 and 37 °C. As depicted in Table 1, the distribution of the amino acid decarboxylase capability differed among the strains and species. In fact, PHE, LYS, and HIS decarboxylating yeasts were less abundant than TYR and ORN degraders.

In particular, the strains belonging to *S. sorbosivorans* (2), *T. pretoriensis* (2), and *T. delbrueckii* (2) did not show any decarboxylase activity against the amino acids tested. On the contrary, the main amino acid decarboxylating yeasts were *S. cerevisiae* (22/38) and *Pichia kudriavzevii* (20/54), and the latter species was the only one showing His decarboxylase activity. In addition, the strains of *P. manshurica* (3/13) and *Z. bisporus* (1/2) were able to decarboxylate only Orn, while the strains of *S. cerevisiae* (22/38) and *T. asahii* var. *asahii* (3/3) showed decarboxylase activity against all amino acids except to His. With regards to *C. parapsilosis* (6/10) *and W. anomalus* (6/6)*,* both were able to decarboxylate three different amino acids (Tyr, Phe and Orn). In the same way, 7/8 strains of *H. burtonii* were able to decarboxylate Tyr, Orn, and Lys. On the other hand, *S. pombe* was positive for the decarboxylation of two amino acids (Tyr and Orn).

To explore the possible strain discrimination according to their decarboxylase activities, a heatmap analysis was further conducted, considering only the positive strains. As evidenced in Figure S1, strong (scale 3) Orn decarboxylase activity was a very diffuse trait among *P. kudriavzevii, H. burtonii,* and *S. cerevisiae* positive strains, while a moderate activity (scale 2) for Tyr and Orn was present in *C. parapsilosis* and *W. anomalus.*

### 3.2. Simulated Fermentation Using Cocoa Pulp Medium (SCPM)

In this part of the study, we simulated cocoa beans fermentation to investigate decarboxylation activity in situ. Samples were subjected to different temperatures from 25 °C at the beginning of the fermentation, reaching a maximum of 46 °C from 48 to 120 h of fermentation. For this, we selected the strains *P. kudriavzevii* ECA33 and *S. cerevisiae* 4, which did not show decarboxylase activity of the amino acids tested individually in this study. These strains were characterized previously by their good thermotolerance, as well as a strong pectinolytic and β-glucosidase activity in a synthetic medium [18] suggesting that they may have an important impact on cocoa production.

#### 3.2.1. Microbial Growth and pH Dynamics

The trend of the yeast growth in the lab-scale cocoa beans fermentation was similar. In fact, they increased during the first 24 h, in which the temperature increased from 25 to 30 °C, and successively, with the time extension and temperature increase, the cell counts decreased markedly in all three batches. However, significant differences (*p* < 0.05) were observed among the samples inoculated with *S. cerevisiae* 4 (SCB) and *P. kudriavzevii* ECA33 (PKB), with respect to the control samples overall after 24 h of incubation.

**Figure 1:**
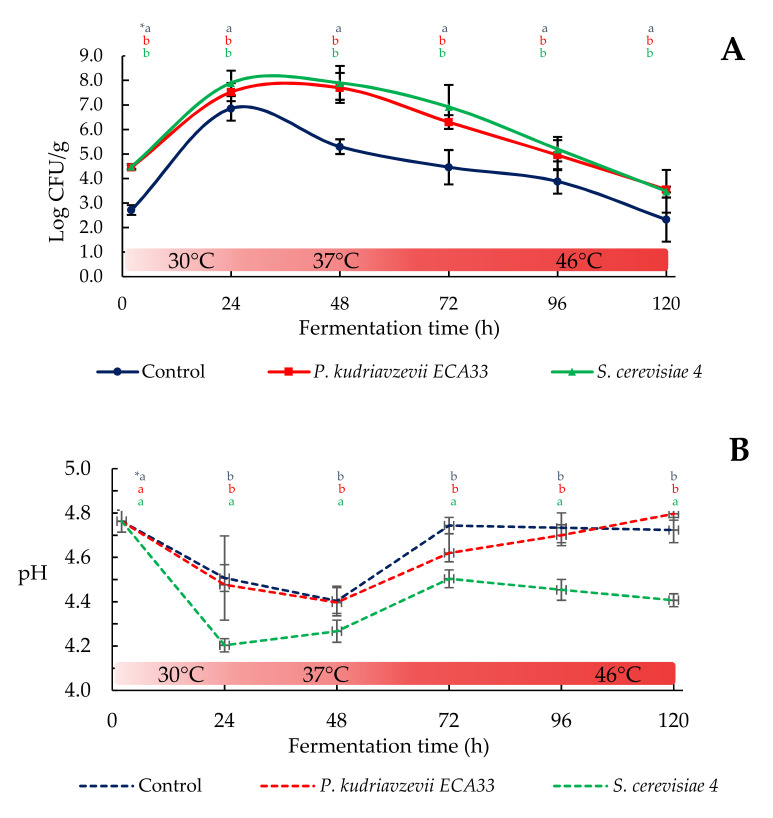
(**A**): Growth dynamics of the total yeasts during the simulated fermentation of re-hydrated cocoa beans. Batch inoculated with *P. kudriavzevii* ECA33 (red line), batch inoculated with *S. cerevisiae* 4 (green line) and uninoculated control batch (blue line). (**B**): pH variation during the simulated fermentation of re-hydrated cocoa beans. Batch inoculated with *P. kudriavzevii* ECA33 (red dashed line), batch inoculated with *S. cerevisiae* 4 (green dashed line) and uninoculated control batch (blue dashed line). * Different letters in the same column (hour) indicate significant differences (*p* < 0.05) among inoculated strains; first row Control, second row *P. kudriavzevii* ECA33, and third row *S. cerevisiae* 4.

As evidenced in Figure 1A, while in the first 24 h when the temperature was changed from 25 to 30 °C, all the different batches showed an increase in the yeast counts of about 4 Log CFU/g, after this period and with the temperature increase at 37 °C, there was a remarkable decrease of the total yeasts (2.5 Log CFU/g) in control samples, indicating probably the low proportion of thermotolerant yeast. On the contrary, in the inoculated batches, the counts remained constant. After this period and with the increase of the temperature to 46 °C, a significant reduction of about 4.3 Log CFU/g was observed for both PKB and SCB, and 5.5 Log CFU/g for control batch (CB), at 120 h. From our data, it is possible to hypothesize that the maximum growth for the *P. kudriavzevii* and *S. cerevisiae* was reached at 24 h, but further studies using molecular techniques will be performed to confirm this hypothesis. Other authors [26,27] have reported different growth patterns of *P. kudriavzevii*, as they stated that it achieved the maximum growth values at 96 h of fermentation, after inoculating 5.5 Log CFU/g. In particular, these authors found that *P. kudriavzevii* inocula achieved counts that were 2 Log higher (over 8.9 after 96 h fermentation) when compared to spontaneous fermentation in CB (6.8 Log CFU/mL at 96 h fermentation).

At the beginning of fermentation, the presumptive LAB was detected at levels of 3.9 ± 0.12 Log CFU/g. After 24 h, in batches, CB and PKB, their cell density increased to 6.2 ± 0.21 Log CFU/g, while slightly higher counts were detected in samples SCB (6.84 ± 0.35 Log CFU/g). LAB reached the maximum growth at 48 h with similar cell counts (7.5 ± 0.28 Log CFU/g for CB and PKB to 7.7 ± 0.25 Log CFU/g for SCB). The cell density in all the batches stayed relatively stable until 72 h, and afterwards decreased reaching counts of 4.8 ± 0.36 Log UFC/g for CB, and 5.5 ± 0.26 Log UFC/g and 5.8 ± 0.29 Log UFC/g for PKB and SCB respectively, at the end of fermentation (120 h).

The AAB were under the detection limit at the beginning of fermentation but increased after 24 h as a function of time, with differences among the batches. In fact, at 24 h they reached very low values of 2.1 ± 0.28 Log CFU/g in the CB and 1.5 ± 0.16 and 3.7 ± 0.27 Log CFU/g for PKB and SCB, respectively. Comparable microbial population dynamics were observed in the three batches, as this microbial group was steadily increasing from 48 to 72 h reaching counts of 5.7 ± 0.30, 5.2 ± 0.29, and 6.7 ± 0.36 Log UFC/g, respectively for the CB, PKB, and SCB, at 72 h of fermentation. After that, they decreased and at the end of fermentation, they reached 2.9 ± 0.21 Log UFC/g for CB and PKB and 3.2 ± 0.46 Log UFC/g for SCB samples.

In general, the pH varied according to the fermentation process and strain added to the cocoa beans batch (Figure 1B). In particular, during the first 24 h at 30 °C, *S. cerevisiae* 4 inoculation caused a significant reduction of pH to 4.20 ± 0.08, which was statistically different (*p* < 0.05) from the control and PKB samples, in which pH value was 4.50 ± 0.07. With the temperature increase at 37 °C for a further 24 h, an additional decrease in the pH values was observed in these batches. The differences were probably due to the different values of LAB and AAB counts. In contrast, during the subsequent fermentation period (from 48 to 120 h), an increase of pH was observed reaching values of around 4.70 ± 0.06 and 4.80 ± 0.02 for the control and PKB samples, which were significantly different from the samples SCB 3 (4.40 ± 0.02). While the decrease of pH is related to the production of lactic acid and acetic acid that are produced by the degradation of the sugar present in the pulp by the microorganisms [28], the increase of pH in the different batches could have been due to: (i) the activity of the LAB that converted citric acid into lactic acid and acetic acid, which causes an increase of pH from 3.0 to 4.0, because of the higher pKa values of the latter organic acids compared to citric acid; (ii) the oxidation of acetic and lactic acid by AAB, and; (iii) the increase of basic compounds as well as BAs [29]. In this context, it is well established that during fermentation, proteins are slightly degraded at two days, but proteolysis becomes intense later [30] when the concentrations of free amino acids in the pulp and beans increase about fivefold during fermentation [1].

#### 3.2.2. Changes in Biogenic Amines Content

In our experiment, seven BAs and namely spermine (SPR), spermidine (SPD), tryptamine (TRY), 2-phenylethylamine (PHE), putrescine (PUT), cadaverine (CAD), tyramine (TYR) and histamine (HIS), were detected and quantified during the fermentation time.

**Figure 2:**
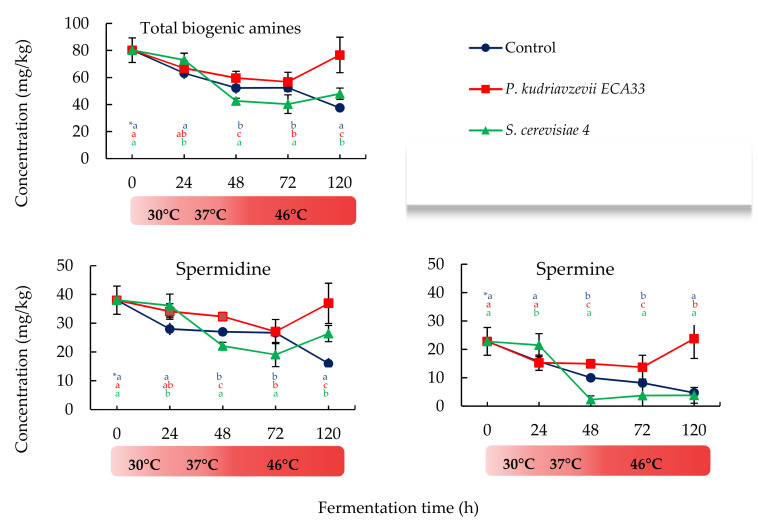
Trends of total biogenic amines, spermidine and spermine in the batches inoculated with *P. kudriavzevii* ECA33 (red line), with *S. cerevisiae* 4 (green line), and in uninoculated control (blue line). Data are expressed as mean ± standard deviation (SD) values from duplicate determinations. * Different letters in the same column (hour) indicate significant differences (*p* < 0.05) among inoculated strains; first row control, second row *P. kudriavzevii* ECA33, and third row *S. cerevisiae* 4.

In general, the samples inoculated with *P. kudriavzevii* ECA33 showed a major accumulation of total BAs (Figure 2), which was correlated to a higher presence of SPD and SPR. The significantly higher levels of both BAs detected in all samples at the beginning of the experimental time decreased markedly during 48–72 h of fermentation. This trend continued until the end of the fermentation time in control batch (CB) samples, while in the samples inoculated with *P. kudriavzevii* ECA33 (PKB) and in those inoculated with *S. cerevisiae* 4 (SCB), a subsequent increase of these polyamines was observed after 72 h, with a major accumulation in PKB samples.

The influence of the inoculated strains on the occurrence of TRY, PHE, PUT, CAD, and TYR content is shown in Table 2, in which it is possible to observe: (i) a significant reduction of TRY content in the first 24 h followed by an overall increase in SCB in which, with the increase of temperature and fermentation time, TRY increased by near 3 folds with respect to CB; (ii) a PHE reduction of (1.6 folds and 6 folds) and lesser reduction of PUT (1.5 and 1.6 folds), respectively for PKB and SCB at 120 h of fermentation time, with respect to CB. These results were unexpected since it is known that amine oxidases have a pH optimum of 7.0 and a pH range of 5–10 [31] and the pH of our samples fluctuated from 4.2 to 4.8 during fermentation; (iii) a significant increase followed by a reduction of CAD content during 24 and 48 h of incubation in both PKB and SCB samples; IV) no effect on TYR content, as in all the three batches, the profile was very similar with a decrease in the content of this BA, thus indicating that the microbiota of the Colombian cocoa samples prevented the accumulation of TYR.

Pearson’s correlation index among the amounts of BAs of the different batches and temperature of fermentation at different times were determined for each BA. As depicted in Table 3, a strong correlation was found between PKB and TRY (ß = 0.89) and PUT (ß = 0.82) content. Similar results were observed for the CB. On the other hand, while TRY was only weakly correlated with the total BA in SCB, TYR was weakly correlated (ß = 0.55 and ß = 0.59, respectively) with both PKB and CB. A strong negative correlation was found in PKB and SCB with CAD (ß = −0.93). Furthermore, a strong negative correlation was found between temperatures (reached in the batches at different fermentation steps) with PUT (ß = −0.68) and TYR (ß= −0.89) content. On the bases of these results, it appears that temperature is important for the expression of enzymes able to degrade TYR and PUT of both inoculated batches with *P. kudriavzevii* ECA33 and *S. cerevisiae* 4.

#### 3.2.3. Organic Acids Dynamics

**Figure 3:**
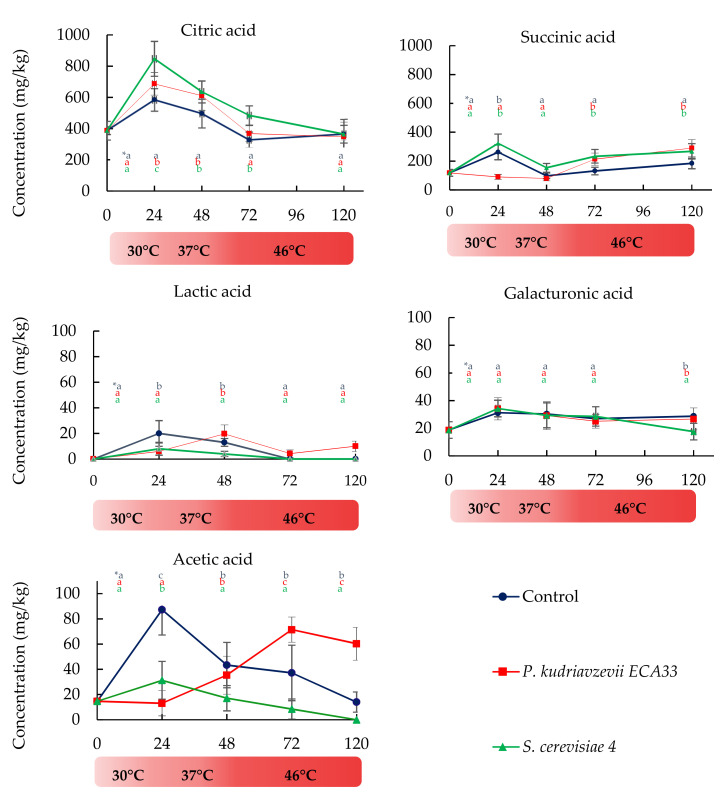
Citric acid, succinic acid, lactic acid, galacturonic acid, and acetic acid production during fermentation of *P. kudriavzevii* ECA33 (red line), *S. cerevisiae* 4 (green line) and uninoculated control (blue line). Data are expressed as mean ± standard deviation (SD) values from duplicate determinations. * Different letters in the same column (hour) indicate significant differences (*p* < 0.05) among inoculated strains; first row control, second row *P. kudriavzevii* ECA33, and third row *S. cerevisiae* 4.

Citric acid was the most abundant organic acid in unfermented cocoa beans, and it was metabolized differently in the three batches (Figure 3). Moreover, an unexpected profile of citric acid was observed, compared to those reported in the literature, in which other authors [32] reported an initial consumption of this acid. In fact, in our experiments, during early fermentation time (the first 24 h), there was an accumulation of citric acid overall in SCB samples, in which the level was 846.8 ± 35.4 mg/kg, correlated to the major decrease of pH values at this experimental time. After 24 h of fermentation, citric acid dropped below 370 mg/kg at the expiration of the fermentation time in all the samples.

Our results revealed that the addition of *P. kudriavzevii* ECA33 and *S. cerevisiae* 4 strains to the cocoa beans induced changes in the microbiota performance in response to temperature variations. Hence, the maximum of lactic and acetic acid production was reached at 24 °C for SCB samples and at 48 h (lactic acid) and 72 h (acetic acid) for PKB samples. On the other hand, *P. kudriavzevii* ECA33 that is more thermotolerant than *S. cerevisiae* 4 [18], promoted a major accumulation of both lactic (19.7 ± 0.9 mg/kg) and acetic acid (71.5 ± 3.6 mg/kg), with respect to SCB and CB samples, at the end of the fermentation period.

In addition to citric acid, succinic acid was also present at high concentrations in raw cocoa beans (118.3 ± 5.9 mg/kg). Yeasts produce succinic acid by sugars fermentation, together with ethanol. In our experiment, the evolution of succinic acid was similar in the CB and SCB samples, in which there was an increase during the first 24 h, followed by a decrease in the next 24–48 h; successively, there was a significant increase until the end of fermentation. On the contrary, in the PKB samples, the succinic acid concentration remained almost constant during the first 24 h, and after that, it increased remarkably for all the rest of fermentation.

Galacturonic acid derives from the pectinolytic activity of yeasts, fundamental for the degradation of cocoa bean pulp. At the beginning of the fermentation, the value of galacturonic acid was 18.7 ± 0.9 mg/kg, and its accumulation was very similar in all the samples during fermentation. However, a slight difference at the end of fermentation was observed for SCB samples, reaching 17.6 ± 0.8 mg/kg values, which were lower than those observed in PKB and CB samples.

#### 3.2.4. Volatile Compounds

In our experiment, the most abundant VOCs obtained from the different fermented cocoa beans batches are grouped into the following four chemical families: alcohols, aldehydes, ketones, and esters (Figure S2). In particular, alcohols followed by esters were the most abundant VOCs; these compounds have been associated with the yeast metabolism. In addition, ketones were the group of compounds that underwent the biggest changes as a result of fermentation time.

**Figure 4:**
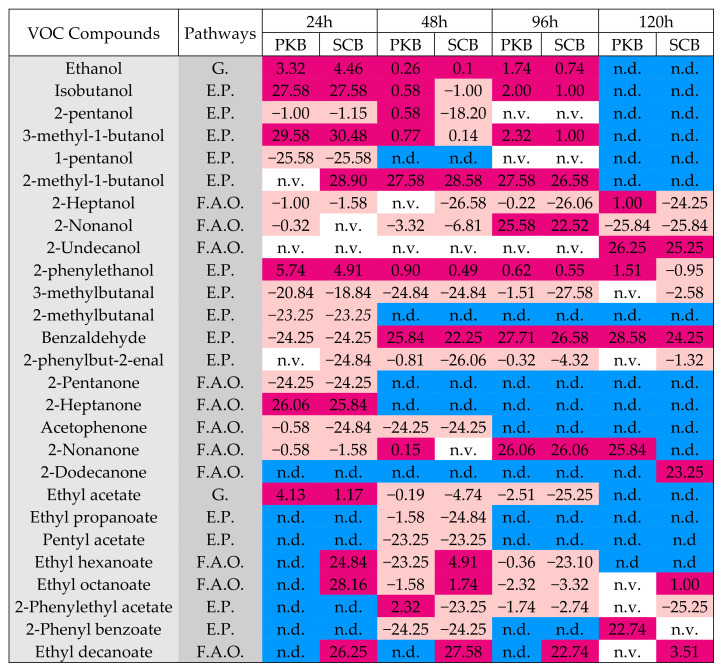
Combination of charts showing altered volatile organic compounds (VOCs) and addressed microbiota metabolic pathways, where numbers inside boxes display the base 2 logarithms of fold-change calculated with respect to the response in uninoculated samples; blue color (n.d.) = not detected during fermentation in all batches; white color (n.v.) = no variation with respect to control; red color (+) = increase after addition of the strains; pink color (−) = decrease after addition of the strains. Pathways: F.A.O.: Fatty Acids Oxidation. G.: Glycolysis. E.P.: Ehrlich Pathway.

Figure 4 gathers the data regarding changed VOCs and addressed microbiota metabolic pathways, where numbers inside the boxes display the base 2 logarithms of fold-change calculated with respect to the response in the untreated batch. At the beginning of the fermentation (24 h), in both inoculated batches, there was an induction to the production of alcohols deriving from the glycolysis and Ehrlich pathway, as observed by the production of ethanol, 2-methyl-1-propanol, 2-methyl-1-butanol, 3-methyl-1-butanol, and 2-phenylethanol. At the same time, a decrease in aldehydes and ketones accumulation was also observed. While the major production of ethanol could be due to the major activity of pectinolytic enzymes and the fermentation of pulp sugars by the added yeast, the accumulation of branched-chain alcohols could be related to the major presence of the aldehyde dehydrogenase enzymes in the bulk, which could be produced by LAB and in major quantities by yeasts. In fact, a reduction of the correspondent branched-chain aldehydes was observed. Although the accumulation of ethanol increased significantly in the inoculated samples during the first 24 h, with respect to uninoculated control, the peak was reached at 48 h in the three batches. At the end of fermentation, ethanol was not detected.

While 2-phenylethanol was detected during the whole process, with the highest amounts in PKB samples, 3-methyl-1-butanol was observed at 24 h, and then its peak area decreased, and after 48 h and at the end of the fermentation, this compound was not detected probably due to chemical degradation or volatilization [33]. On the other hand, whereas we observed in both SCB and PKB samples a strong reduction of 3-methylbutanal, we detected accumulation of 2-methylbutanal that derives from leucine and isoleucine deamination by leucine aminotransferase produced by yeasts, as well as accumulation of benzene acetaldehyde that derives from the oxidative phenylalanine deamination by phenylalanine aminotransferase produced by yeasts, conferring a green odour. Moreover, a significant increase of benzaldehyde (bitter, almond, grass flavours) deriving from phenylalanine was observed in both batches during the fermentation time, especially in PKB samples. Finally, we detected an early production of esters, and namely ethyl acetate, ethyl hexanoate, ethyl octanoate, and ethyl decanoate, in SCB samples with respect to PKB and CB samples.

With time and temperature increase, differences in VOCs profiles were more evident among the different batches. These differences were particularly related to the major accumulation of alcohols and esters in PKB samples at 48 h of fermentation, and overall, of 2-methyl-1-propanol, 1 pentanol, 3 methyl-1-butanol, 2-methyl-1-butanol, phenyl ethyl alcohol, and 2-phenylethyl acetate. During the same period, SCB samples continued to show the highest amounts of the esters ethyl octanoate, ethyl hexanoate and ethyl decanoate. In addition, we observed a significant increase of benzaldehyde in both inoculated batches.

At the end of fermentation, in PKB samples, fatty acid and amino acid-derived VOCs prevailed, with a major presence of the 2-nonanone (flowery fatty flavors), 2-phenyl benzoate (sweet, rose, honey-like/floral taste), 2-phenylethanol, and 2-heptanone (floral and fruity flavors). SCB samples contained more VOCs deriving from fatty acid metabolism such as methylketones and their secondary alcohols, identified as fruity volatiles. In fact, 2-dodecanone, ethyl octanoate (fruity banana, apricot, brandy flavors), and ethyl decanoate (pear, grape) were the most abundant compounds.

## 4. Discussion

It is well known that the use of selected autochthonous starter cultures consisting of yeast, LAB, and AAB is a suitable option to improve the cocoa bean fermentation process. In this study, the high number (68/145) of yeast strains able to decarboxylate at least one of the amino acids tested suggests that they could contribute to the BA accumulation during cocoa fermentation. However, the production of BAs by decarboxylase positive strains, here screened in vitro, does not necessarily imply the same behaviour in cocoa fermented beans, since BA accumulation is affected by various factors (temperature, pH, accessibility of precursor amino acids, temperature/time of fermentation, among other factors) and their interactions [34]. In any case, the capability to form BAs of *C. parapsilosis, H. burtonii*, *P. kudriavzevii*, *S. pombe*, *T. asahii* var. *asahii*, *W. anomalus, and Z. bisporus* is very interesting, as it was not reported before. These results suggest that non-*Saccharomyces* yeasts, especially *P. kudriavzevii* and *T. asahii* var. *asahii*, are suitable reservoirs of a wide amino acid decarboxylases portfolio. In the literature, there are few studies on the formation of BAs by yeasts, and a great part of them are related to *S. cerevisiae* strains or other non-*Saccharomyces* yeasts isolated from wine. For example, the ability of *S. cerevisiae* to decarboxylate amino acids and produce TYR and HIS during alcoholic fermentation of grape juice has been reported by [35]. In the same way, previous studies have demonstrated that *P. manshurica* strains were able to synthesize HIS and CAD in wine [36,37]. The amino acid decarboxylation activity has been reported also for *Debaryomyces hansenii* CLIB 3084, isolated from palm wine [38].

In our study, cocoa pulp simulation medium was useful to compare the modification induced by *P. kudriavzevii* ECA33 and *S. cerevisiae* 4 to the cocoa beans during fermentation in terms of BAs, organic acids, and VOCs production. With regards to BAs accumulation, our results pointed out that although during fermentation spermidine content was significantly reduced, it became the predominant bioactive amine during the fermentative process. In line with our results, Do Carmo brito et al. [9] reported that this BA contributed from 38 to 55% to the total of BAs in cocoa beans during fermentation. The polyamines SPE and SPD are constitutive compounds of eukaryotic cells, as essential polycations involved in the regulation of cell proliferation [39]; thus, their accumulation could be related to their occurrence in pods and microorganisms of cocoa pulp medium, in both inoculated and uninoculated samples.

During fermentation, a switch-off and switch-on of Bas content with the increase of temperature and fermentation time was observed, likely due to the balance between amino acid decarboxylase and amine oxidase enzymes activity, which cause accumulation or degradation of Bas, respectively. The amine oxidase activity can be considered an alternative source of energy for the microorganism, in conditions of nutritional stress or when other microorganisms compete for the same substrate. Since we did not sterilize the beans before fermentation, it has to be taken into account that other microorganisms involved in cocoa beans fermentation could also play a role in the amino oxidase activity that could be activated with the increase of temperature and/or incubation time. In this context, recently, starter cultures of *Bacillus* capable of degrading and/or incapable of producing Bas have been investigated in fermented foods [40]. In addition, strains of *Brevibacterium, Lactobacillus*, *Pediococcus* spp [41], and *Staphylococcus xylosus* [42] were able to reduce the BAs content.

We have demonstrated that temperature contributes to the switch-off and switch-on of the amines metabolism during simulated cocoa fermentation, by determining different accumulation profiles in all the batches at the end of the fermentation, depending on the inoculated yeast. These changes can exert positive and negative effects, for example: (i) a major polyamine (SPD and SPR) accumulation, especially in PKB samples, is considered by some authors as a desirable treat for the antioxidant activity, which is beneficial for products shelf life and human health [43]; (ii) a significant reduction of PHE and CAD, overall in SCB samples with respect to control, is an interesting result because high levels of β-phenylethylamine found in foods cause hypertension, headaches, vomiting, and perspiration (EFSA, 2011); (iii) a significant increase of TRY, and PUT; (iv) a significant decrease of TYR in all the batches during fermentation time. TRY plays an important role in the transport of long-chain fatty acids into mitochondria for oxidation and in the synthesis of choline. Whitfield and Large [44] observed that ascosporogenous yeasts produced monooxygenases for dimethylamine and trimethylamine that were significantly more stable than the corresponding enzymes found in *Candida utilis*. The reduction of TRY is particularly interesting since it could be even more cytotoxic than TYR and even HIS [45].

On the other hand, the organic acids profile has important implications for the process and chocolate quality [2]. The accumulation of organic acids reported here is the result of the balance between consumption and production by the different microbial groups present in the fermented cocoa seed mass and overall LAB, AAB, yeasts, and other less-studied microbial groups like *Enterobacteriaceae* and *Bacillus* spp. In our experiment, the addition of a significant proportion of *S. cerevisiae* and *P. kudriavzevii* may have influenced the microbial successions and consequently the end products of fermentation. The significant increase of citric acid content during the first 24 h of fermentation observed here could be due to the hydrolytic activity of microorganisms that causes a higher release of citric acid from cocoa beans. Further analyses are needed to confirm this hypothesis. On the other hand, the breakdown of this acid is assumed to modulate the pH of the fermenting cocoa pulp and influence microbial growth. In fact, citrate-positive LAB species, use citric acid as a co-substrate and can decrease the acidity of the cocoa beans. In addition, *Enterobacteriaceae* are also able to assimilate citric acid during cocoa fermentation [46]. Yeasts utilize also pulp citric acid, as reported for *Candida kruseii* by [47]. Using metagenomics analysis, Illeghems et al. [48] demonstrated that citrate assimilation and pectinolysis have been associated with *Enterobacteriaceae*.

A question can be raised regarding the lower accumulation of acetic and lactic acid in SCB samples, with respect to CB and PKB samples. This suggests that the environmental conditions that *S. cerevisiae* 4 recreated were enough probably to: (i) reduce production of acetic acid by *Enterobacteriaceae*, LAB and AAB, as it happens when heterofermentative LAB are exposed to high ethanol concentration [49]; (ii) generate changes in the AAB population, since some AAB species differ in the speed with which they can oxidize ethanol and lactic acid simultaneously, and in fact, species belonging to the genus *Acetobacter* can over-oxidize acetic and lactic acids into CO_2_ and H_2_O, decreasing cocoa beans acidity [12], *Acetobacter pasteurianus* can fast oxidize lactic acid to acetoin [50], and, as reported by Mansour et al. [51], yeasts like *Yarrowia lipolytica, Debaryomyces hansenii, Kluyveromyces lactis*, and *Kluyveromyces marxianus* can catabolise lactic acid; (iii) increase the activity of esterases, hemiacetal dehydrogenases (HADHs), or alcohol acetyltransferases responsible for the conversion of acetic acid to ethyl acetate in yeasts [52] present in the cocoa bulk, as evidenced in Figure 4 for SCB samples.

In mixed cultures, the microbial interactions occur by means of several mechanisms, and their effects on the strains’ fitness involved may either be positive, neutral, or negative [49]. In our experiment, we observed that the addition of autochthonous *P. kudriavzevii* and *S. cerevisiae* strains increased the synthesis of VOCs, which has effects on the cocoa fermented beans’ flavor. In fact, the diverse VOC levels differentiated the control batches from the SCB and PKB, which emphasized their complementary enzymatic activity. The high levels of branched alcohols, which derive from branched amino-acid catabolism, aldehydes, and ketones, may be the consequence of an accelerated liberation of free amino acids precursors in SCB and PKB samples. In particular, 2-phenylethanol and 3-methyl-1-butanol that derive from the dehydrogenation of the correspondent aldehydes, (3-methyl-1-butanal and 2-phenylbut-2-enal respectively), are considered as key-aroma markers of fermented cocoa beans and reported as desirable in cocoa bean fermentation for high quality [22], because they both confer honey, caramel, sweet, chocolate flavor. On the other hand, we detected an early production of the esters in SCB samples with respect to PKB and CB samples, which could be linked to the yeast metabolism. These compounds represent the typical aroma components in unroasted fermented cocoa beans, and they arise from amino acids [33] or in the case of ethyl acetate from esterase, hemiacetal dehydrogenases (HADHs), or alcohol acetyl-transferases responsible for the conversion of acetic acid to ethyl acetate in yeasts, as mentioned above.

At the end of fermentation, the aromatic profile of the two inoculated batches was very different, and this may be explained by the differences in utilization of the flavor precursors by the microbiota of the two batches. In fact, while fatty acid and amino acids-derived VOCs prevailed in PKB samples, in particular, 2-nonanone (flowery fatty flavor), 2-phenyl benzoate (sweet, rose, honey-like/floral taste), 2-phenylethanol, and 2-heptanone (floral and fruity flavors), in SCB samples, we found mainly VOCs deriving from fatty acid metabolisms such as methylketones and their secondary alcohols, which are usually identified as fruity volatiles. In fact, 2-dodecanone, ethyl octanoate (fruity banana, apricot, brandy flavors) and ethyl decanoate (pear, grape) were the most abundant compounds in SCB samples. These results confirm the suitability of selected yeast strains to produce fermented cocoa beans with different aroma compounds that positively affect the sensory profile of cocoa.

## 5. Conclusions

In this study, for the first time, we report a high intraspecific variability in amino acid decarboxylase activity of yeasts isolated from Colombian cocoa beans. Some of these strains, in particular, *S. cerevisiae* 4 and *P kudriavzevii* ECA33, showed interesting technological properties, revealed by their ability to induce favorable changes in the cocoa bulk and specifically on BAs degradation and VOCs production, as a result of an energy-efficient growth and of the aptitude to react to a changing environment. Although a lab-scale fermentation does not represent fermentation in industrial conditions, our data highlight a significant contribution of temperature and fermentation time to the decarboxylase and amine oxidase activities, leading to reduction, inhibition, or increase of BAs in the cocoa fermentation mass. These activities are crucial both for the quality of the product, in particular for the formation of taste and flavor, and for food safety, considering the potential toxicity of BAs.

One of the most interesting technical features of *S. cerevisiae* 4 was the capability of promoting BAs degradation during cocoa beans fermentation. In addition, our findings pointed out that *P. kudriavzevii* ECA33 induced a major production of fatty acid- and amino acid-derived VOCs, while *S. cerevisiae* 4 induced more VOCs deriving from fatty acids metabolism. Our findings confirm the complexity of the cocoa beans microbiota, whose metabolism can affect both sensory properties and safety of cocoa products.

## Figures and Tables

**Table 1:** Percentage of strains with amino acid decarboxylase activities, showed in vitro by the different yeast species isolated from fermented cocoa and dried beans. Tyr: tyrosine; Phe: phenylalanine; Orn: ornithine; His: histidine; Lys: lysine.

	N° of Total Strains	Tyr	Phe	Orn	His	Lys
*Candida parapsilosis*	10	50	10	60	0	0
*Hydropichia burtonii*	8	37.5	0	62.5	0	12.5
*Pichia kudriavzevii*	54	12.96	15.42	90.74	2.20	90.74
*Pichia manshurica*	13	0	0	23.07	0	0
*Saccharomyces cerevisiae*	38	23.68	5.26	28.94	0	23.68
*Schizosaccharomyces pombe*	4	25	0	25	0	0
*Starmerella sorbosivorans*	2	0	0	0	0	0
*Torulaspora delbruekii*	2	0	0	0	0	0
*Torulaspora pretoriensis*	2	0	0	0	0	0
*Trichosporon asahii* var. *asahii*	3	100	23.07	23.07	0	15.38
*Wickerharmomyces anomalus*	6	100	16.66	83.33	0	0
*Zygosaccharomyces bisporus*	3	0	33.33	0	0	0

**Table 2:** Dynamics of the biogenic amines during cocoa beans simulated fermentation with single cultures of *P. kudriavzevii* ECA33 and *S. cerevisiae* 4.

	Biogenic Amines Content (mg/kg)				
Fermentation Time (h)	0	24	48	96	120
**Control**					
TRY	4.8 ± 0.9 cA	2.6 ± 0.4 bA	0.9 ± 0.1 aA	3.8 ± 0.5 bcA	2.5 ± 0.3 bA
PHE	2.6 ± 0.5 aA	6.3 ± 0.5 bA	6.7 ± 1.9 bA	6.1 ± 1.3 bA	6.2 ± 0.3 bA
PUT	9.0 ± 0.6 cA	6.3 ± 0.7 bA	3.8 ± 0.6 aA	3.7 ± 0.0 aA	4.7 ± 0.6 abA
CAD	0.8 ± 0.1 aA	3.4 ± 0.5 bA	2.9 ± 0.2 bA	3.3 ± 0.3 bA	3.1 ± 0.8 bA
TYR	2.2 ± 0.1 cA	1.1 ± 0.2 bA	1.0 ± 0.1 bA	0.6 ± 0.1 aA	0.4 ± 0.0 aA
***P kudriavzevii* ECA33**					
TRY	4.8 ± 0.9 cA	4.6 ± 0.1 bcB	3.6 ± 0.1 abB	4.3 ± 0.1 bcA	4.6 ± 0.5 bcB
PHE	2.6 ± 0.5 aA	2.4 ± 0.2 aB	1.9 ± 0.0 aB	3.8 ± 0.1 bB	3.9 ± 0.8 bB
PUT	9.0 ± 0.6 bA	8.6 ± 0.5 bB	5.5 ± 1.1 aB	5.8 ± 1.2 aB	5.7 ± 0.3 aA
CAD	0.8 ± 0.1 aA	0.6 ± 0.1 aB	2.3 ± 0.4 cA	1.5 ± 0.3 bC	1.5 ± 0.2 bB
TYR	2.2 ± 0.1 dA	1.4 ± 0.0 cA	1.0 ± 0.2 bA	0.6 ± 0.1 aA	0.4 ± 0.1 aA
***S. cerevisiae* 4**					
TRY	4.8 ± 0.9 abA	1.7 ± 0.1 aA	5.8 ± 1.1 bC	6.2 ± 1.0 bB	7.2 ± 1.9 bC
PHE	2.6 ± 0.5 bcA	1.6 ± 0.1 abC	3.2 ± 0.8 cC	2.4 ± 0.2 bcC	1.0 ± 0.1 aC
PUT	9.0 ± 0.6 cA	7.8 ± 1.6 cB	7.7 ± 0.7 cA	6.8 ± 1.2 aB	7.4 ± 0.0 bC
CAD	0.8 ± 0.1 aA	2.8 ± 0.4 bA	0.8 ± 0.2 aC	1.0 ± 0.1 aC	1.3 ± 0.1 aB
TYR	2.2 ± 0.1 cA	1.3 ± 0.2 bA	0.8 ± 0.05 aA	1.1 ± 0.1 abC	0.8 ± 0.1 aB

Results are expressed as means ± standard deviations. Different lowercase letters in the same row indicate significant differences (*p* < 0.05) among the fermentation days. Different capital letters in the same column indicate significant differences (*p* < 0.05) among the fermentation batch. TRY: tryptamine; PHE: 2-phenylethylamine; PUT: putrescine; CAD: cadaverine; TYR: tyramine.

**Table 3:** Pearson Correlation Coefficient * of each amine against total biogenic amines (Bas) and temperature, found in model cocoa fermentation systems.

Variable	TRY	PHE	PUT	CAD	TYR	Total BA
Control	0.70	−0.56	0.78	−0.35	0.55	1.00
*P. kudriavzevii* ECA33	0.90	0.21	0.82	−0.93	0.60	1.00
*S. cerevisiae* 4	0.63	0.51	0.43	−0.93	0.35	1.00
Temperature	0.15	0.23	−0.68	0.26	−0.89	−0.41

* Significant at the 0.05 probability level.

## Supplementary Materials

The following are available online at https://www.mdpi.com/2076-2607/9/1/28/s1, Figure S1. Heatmap of correlation between yeast strains and amino decarboxylase activity showed after 8 days of incubation in synthetic medium at 30 °C. Figure S2. The main abundant volatile compound (VOCs) obtained from the heap fermented cocoa beans. Scale expressed as the logarithm to base 10 of the areas.
