# Supplementary material for: Exploring the Capability of Yeasts Isolated from Colombian Fermented Cocoa Beans to Form and Degrade Biogenic Amines in a Lab-Scale Model System for Cocoa Fermentation
Source: Microorganisms. 2020 Dec 24;9(1):28. doi: 10.3390/microorganisms9010028 (PMC7823927; doi:10.3390/microorganisms9010028)
Supplement: Supplementary file 1 [file microorganisms-09-00028-s001.zip › Figure S1.docx]

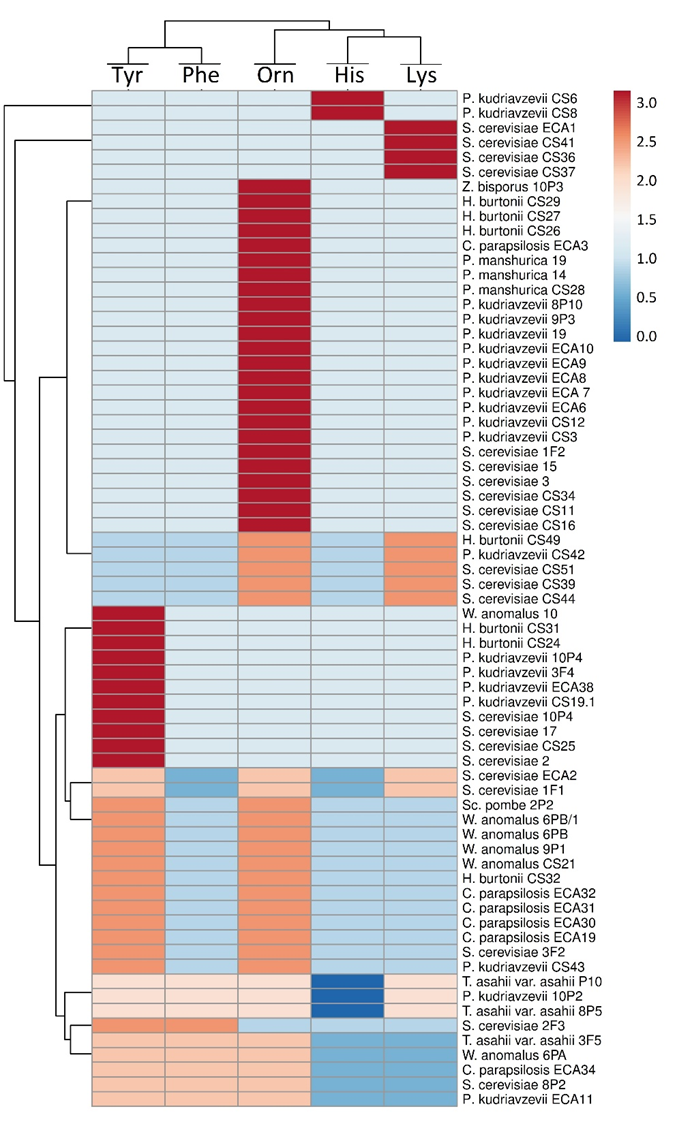


**Figure S1.** Heat map of correlation between yeast strains and amino decarboxylase activity showed after 8 days of incubation in synthetic medium at 30°C.
